# Supplementary material for: Splice-Junction-Based Mapping of Alternative Isoforms in the Human Proteome
Source: Cell Rep. Author manuscript; Available in PMC 2020 Jan 15. (PMC6961840; doi:10.1016/j.celrep.2019.11.026)
Supplement: 2 [file NIHMS1546469-supplement-2.zip › DF1/PXD010154/Esophagus/Esophagus_12_PPP1R12A_ISEMEEELKR.pdf]

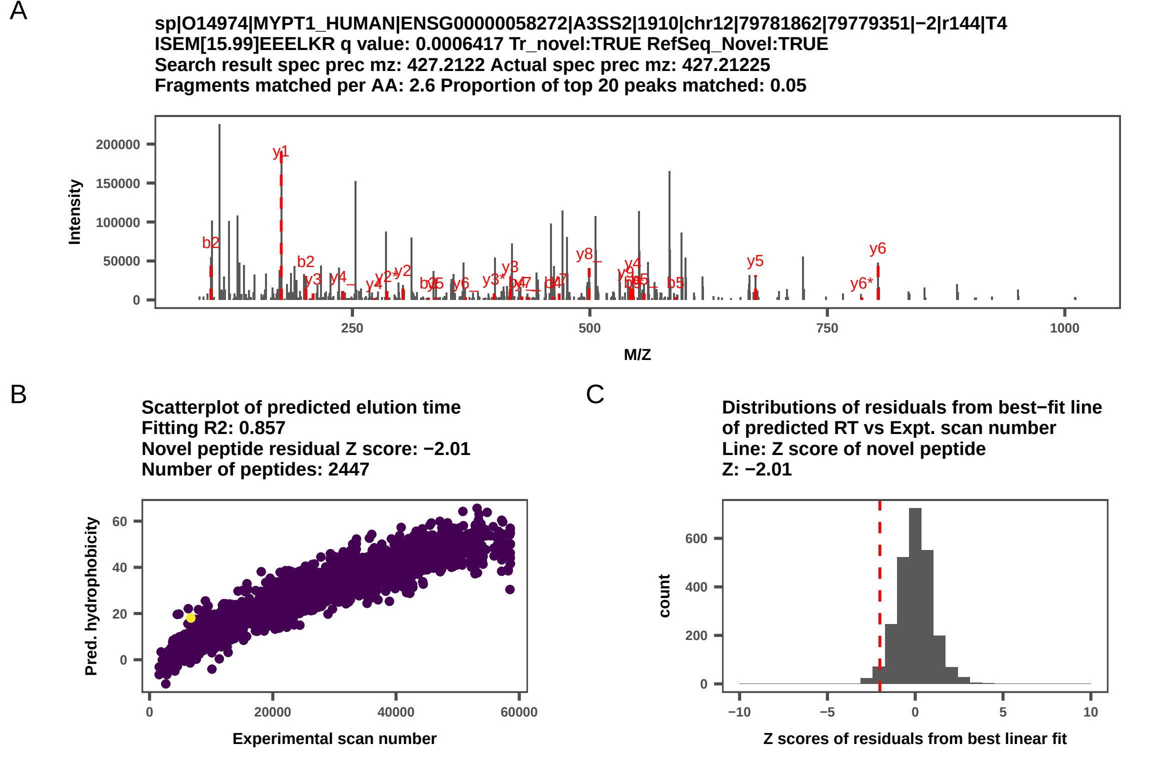
A
sp|O14974|MYPT1_HUMAN|ENSG00000058272|A3SS2|1910|chr12|79781862|79779351|−2|r144|T4
ISEM[15.99]EEELKR q value: 0.0006417 Tr_novel:TRUE RefSeq_Novel:TRUE
Search result spec prec mz: 427.2122 Actual spec prec mz: 427.21225
Fragments matched per AA: 2.6 Proportion of top 20 peaks matched: 0.05
Intensity
200000
150000
100000
50000
0
250
500
750
1000
M/Z
B
Scatterplot of predicted elution time
Fitting R2: 0.857
Novel peptide residual Z score: −2.01
Number of peptides: 2447
Pred. hydrophobicity
60
40
20
0
20000
40000
60000
Experimental scan number
C
Distributions of residuals from best−fit line
of predicted RT vs Expt. scan number
Line: Z score of novel peptide
Z: −2.01
count
600
400
200
−10
−5
5
10
Z scores of residuals from best linear fit
